# A systematic review of the protein composition of whole saliva in subjects with healthy periodontium compared with chronic periodontitis

**DOI:** 10.1371/journal.pone.0286079

**Published:** 2023-05-24

**Authors:** Ana G. Sánchez-Medrano, Rita E. Martinez-Martinez, Ruth Soria-Guerra, Diana Portales-Perez, Horacio Bach, Fidel Martinez-Gutierrez

**Affiliations:** 1 Facultad de Estomatologia, Universidad Autonoma de San Luis Potosi, S.L.P., Mexico; 2 Facultad de Ciencias Químicas, Universidad Autonoma de San Luis Potosi, S.L.P., Mexico; 3 Centro de Investigación en Ciencias de la Salud y Biomedicina, Universidad Autonoma de San Luis Potosi, S.L.P., Mexico; 4 Division of Infectious Diseases, Department of Medicine, University of British Columbia, Vancouver, BC, Canada; Yerevan State Medical University Named after Mkhitar Heratsi, ARMENIA

## Abstract

**Context:**

Periodontitis is a chronic multifactorial inflammatory disease linked to oral microbiota dysbiosis. This disease progresses to infection that stimulates a host immune/inflammatory response, with progressive destruction of the tooth-supporting structures.

**Objective:**

This systematic review aims to present a robust critical evaluation of the evidence of salivary protein profiles for identifying oral diseases using proteomic approaches and summarize the use of these approaches to diagnose chronic periodontitis.

**Data sources:**

A systematic literature search was conducted from January 1^st^, 2010, to December 1^st^, 2022, based on PICO criteria following the Preferred Reporting Items for Systematic Reviews and Meta-Analyses (PRISMA) guidelines and by searching the three databases Science Direct, Scopus, and Springer Link.

**Study selection:**

According to the inclusion criteria, eight studies were identified to analyze the proteins identified by proteomics.

**Results:**

The protein family S100 was identified as the most abundant in patients with chronic periodontitis. In this family, an increased abundance of S100A8 and S100A9 from individuals with the active disease was observed, which strongly relates to the inflammatory response. Moreover, the ratio S100A8/S100A9 and the metalloproteinase-8 in saliva could differentiate distinct periodontitis groups. The changes in protein profile after non-surgical periodontal therapy improved the health of the buccal area. The results of this systematic review identified a set of proteins that could be used as a complementary tool for periodontitis diagnosis using salivary proteins.

**Conclusion:**

Biomarkers in saliva can be used to monitor an early stage of periodontitis and the progression of the disease following therapy.

## Introduction

According to the 216 Global Health Burden, out of 328 diseases, permanent caries and periodontal disease ranked in positions 1 and 11 among the most prevalent human diseases [1]. Periodontitis is a chronic multifactorial inflammatory disease linked to oral microbiota dysbiosis. A recent global meta-analysis showed that the prevalence of apical periodontitis in at least one tooth was 52% of pooled samples worldwide [2]. Although biofilm formation is the primary etiological factor, other host-related factors, such as genetics, immunologic and environmental factors, are associated with periodontitis, making it challenging to establish its diagnosis in the early stages [3]. This disease progresses to infection that stimulates a host immune/inflammatory response, with progressive destruction of the tooth-supporting structures. Moreover, periodontitis has been strongly associated with systemic diseases such as diabetes, cardiovascular disease, rheumatoid arthritis, and the development of Alzheimer’s disease [4, 5].

Periodontal injury is strongly related to the myeloid cells, which can infiltrate the gingival tissue, destroying alveolar bone due to the production of metalloproteinase (MMP)-12 [6]. More recently, a study reported that in addition to MMP-12, calgranulins are also implicated in innate immune responses [7]. Furthermore, these proteins are members of the S100 family involved in various physiological functions, including calcium regulation, metabolism, cell multiplication, and inflammation [8].

The clinical diagnosis of periodontitis requires the use of a standardized periodontal probe to measure pocket depth (PD) and clinical attachment loss (CAL) by circumferential evaluation of erupted teeth concerning the gingival margin and cement-enamel junction (CEJ) [9]. However, these indices could be subjective because they depend on the pressure applied to insert the periodontal probe in the crevice, the anatomic details of teeth, limitations in the mouth opening, and some discomforts [10]. Therefore, there has been an increasing interest in proposing a diagnostic test using biomarkers that could detect periodontitis at its early stages, the active phases of the disease and monitoring the progression of periodontal therapy. For instance, different types of samples have been used to detect the evolution of periodontal diseases, such as periodontal samples and gingival tissue, where the transforming growth factor-β downregulation had a relationship with tissue destruction [11]. In addition, non-invasive sample like saliva has become a potential source of biomarkers compared to a crevicular fluid due to its easy and rapid collection and higher quantity [12].

Saliva contains organic compounds, exfoliated oral epithelial cells, and microorganisms and may include blood, respiratory secretions, gastric acid from reflux, and food debris [13]. In healthy individuals, saliva is produced from 0.5 to 1.5 L per day [14]. The major salivary glands: parotid, submandibular and sublingual, secrete about 90% of human saliva, and the rest by minor salivary glands located throughout the oral mucosa within a pH from 6.0 to 7.0 [15].

This systematic review aimed to present a robust critical evaluation of the evidence of salivary protein profiles for identifying oral diseases using proteomic approaches and summarize the use of these approaches to diagnose chronic periodontitis.

To address the objective, Participants, Interventions, Comparisons, Outcomes, and Study design (PICOS) criteria were set. Three PICOS questions were formulated according to the Preferred Reporting Items for Systematic Reviews and Meta-analyses (PRISMA) statement guidelines [16].

The questions underpinning this review were as follows (Table 1):

**PICOS question 1.** In healthy individuals over 18 years old, what is the composition and concentration of proteins in the whole saliva compared to chronic periodontitis subjects?

**PICOS question 2.** In patients with periodontitis over 18 years old, which proteins are present in saliva after non-surgical periodontal therapy regarding composition and concentration?

**PICOS question 3.** In adults over 18 years with healthy periodontium or periodontitis, what methods are applied for saliva collection, and what characteristics of the techniques for chemical analysis?

**Table 1:** PICOS criteria.

Criteria	PICOS question 1	PICOS question 2	PICOS question 3
**Population**	Adults ≥18 years with healthy periodontium or periodontitis	Adults ≥18 years with periodontitis	Adults ≥18 years with healthy periodontium or periodontitis
**Intervention**		Subgingival instrumentation (any non-surgical procedures)	
**Comparison**	Subjects with healthy periodontium and chronic periodontitis	Individuals before and after non-surgical periodontal therapy	Saliva collection, quantitative and qualitative chemical analysis methods
**Outcomes**	Composition and concentration of salivary proteins Methods of whole saliva collection and techniques for chemical analysis		
**Study**	Cohort and case–control studies		

## Materials and methods

### Protocol registration and review reporting

The protocol was registered in PROSPERO with ID no. CRD42021220377 and this review was conducted and reported according to the PRISMA statement (S1 Checklist) [16].

### Eligibility criteria

Studies were selected with healthy individuals ≥18 years old and divided based on periodontitis. Subjects excluded from this study were those diagnosed with systemic inflammatory disorders, autoimmune diseases, diabetes mellitus, cancer, intake of antibiotics in the last three months, periodontal therapy in the previous year, and undergoing pregnancy or nursing. Studies were included if they presented a complete report on original research. Ongoing publications, conferences, poster presentations, congresses, meetings, and proceedings were excluded. There were no restrictions on the types of study design eligible for inclusion.

### Search methods

The search used three electronic databases: ScienceDirect, Scopus, and Springer Link from January 1^st^, 2010, to December 31^st^, 2022, including an English language restriction. The search strategy was through combinations of medical subject headings (MeSH) terms and keywords. The search terms were “protein composition of saliva AND periodontal diseases OR chronic periodontitis OR periodontitis chronic OR periodontitis patients OR periodontitis subjects OR periodontal diseases subjects,” “protein composition of saliva AND non-surgical periodontal treatment OR non-surgical periodontal therapy OR subgingival instrumentation OR subgingival debridement OR subgingival scaling OR root planning,” “protein composition of saliva AND healthy periodontium,” “protein composition of saliva AND periodontal inflammation.”

### Study selection

Once the studies were identified by searching the databases, record duplicates were removed and screened by title and abstract by two authors (AGSM and FMG). Eligibility criteria were applied under the PRISMA flow diagram to select the included studies. Disagreement was resolved through discussion or by a third independent reviewer (RESG).

### Data management

Two authors (AGSM and FMG) extracted the data into custom Excel tables, verified by a third author (RESG). Data extracted included demographic information, methodology, and outcomes.

The risk of bias was independently assessed for each study using the Newcastle-Ottawa Scale for Risk of Bias criteria (http://www.ohri.ca/programs/clinical_epidemiology/oxford.asp) for case-control and cohort studies [17]. Discrepancies between the examiners were solved in a consensus, and any disagreement through discussion, if needed, was resolved by RESG.

## Results

### Study selection and characteristics

**Fig 1:**
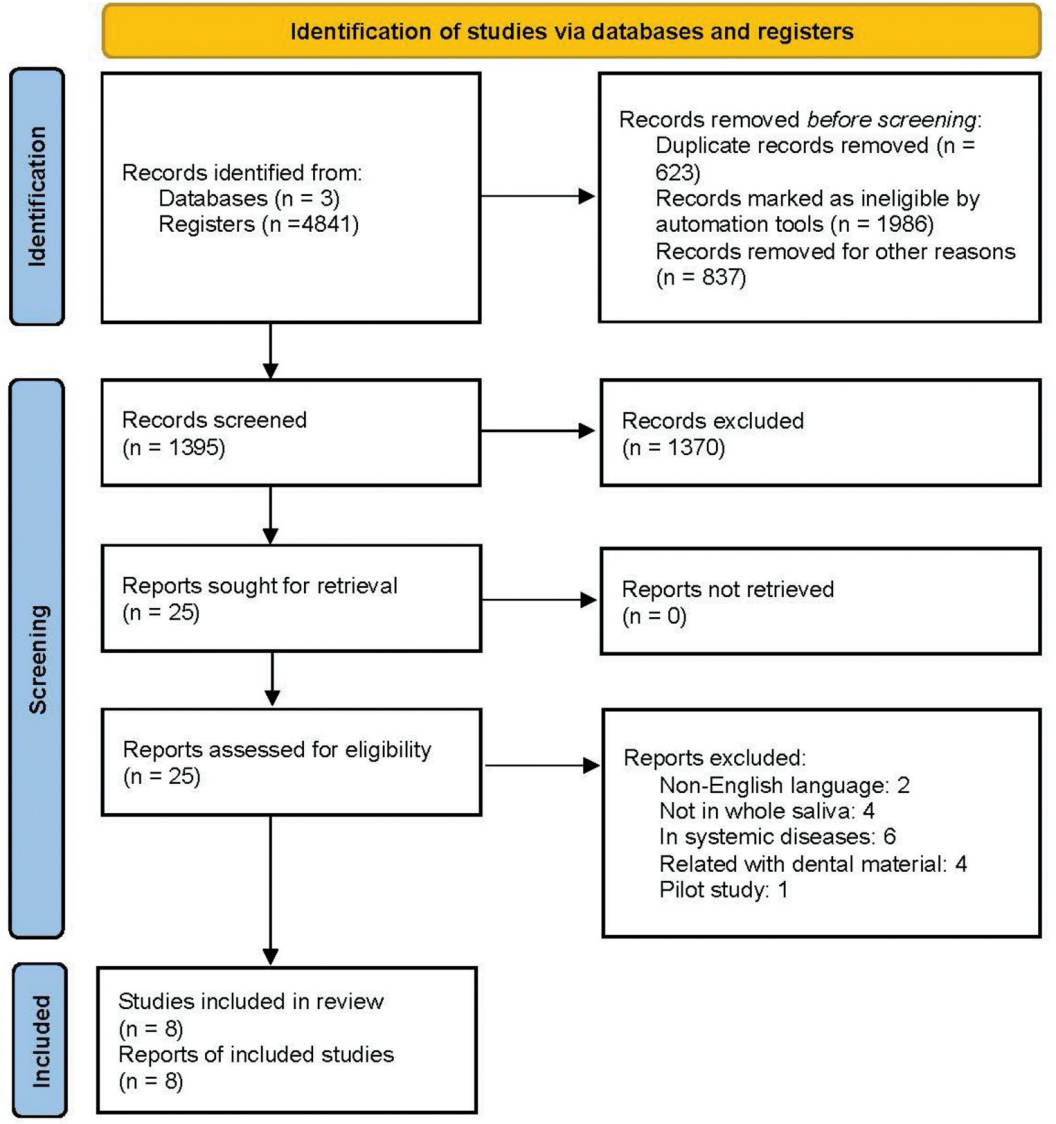
PRISMA flow diagram for studies of literature search and selection [24].

From the search of MeSH terms and keywords combinations, the total number of references obtained was 4841 citations (Fig 1). After the screening, eight studies that met the inclusion were assessed [3, 18–24]. Five of the eight studies focused on the proteomic features of individuals with chronic periodontitis (Table 2). Based on study designs, these eight papers were classified as three studies as case-control [3, 18, 21], and two were cohort studies [19, 20]. The remaining three studies that met the inclusion criteria were related to the anatomic and proteomic features before and after non-surgical periodontal therapy (Table 3) [21–23]. Again, variations in saliva collection methods, definitions of periodontal diseases, and the measurement of protein concentration were found.

**Table 2:** Characteristics and results of included studies with healthy periodontium and chronic periodontitis.

Author, year, country, title	Design and study groups	Definition and measures of periodontal status	Study findings	
			PICO 1	PICO 3
Gonçalves *et al*., 2010 [18] Brazil Comparative proteomic analysis of whole saliva from chronic periodontitis patients.	Case-control study 10 individuals with chronic periodontitis and 10 controls (5 females and 5 males in each group with mean age 45±5.1 and 35.6±9.5, respectively).	BOP, CAL, and PPD. Diagnosis of CP: Six sites BOP on different teeth with PPD ≥6 mm and CAL >5 mm. Control group: <10% of sites BOP and PPD <3 mm.	Values reported: Protein spots Outcomes: 236±10 protein spots for the control and 322±12 for the periodontitis group, which presented 22 exclusive protein spots. Main identified proteins: IgG heavy chain V-II region, serum albumin, α-amylase.	Saliva collection: unstimulated Quantitative and qualitative chemical analysis methods: 2D- gel electrophoresis, liquid chromatography, MALDI-TOF-TOF, and LC-Q-TOF.
Salazar *et al*., 2013 [19] Germany Identification of periodontitis-associated changes in the proteome of whole human saliva by mass spectrometric analysis.	Cohort study 20 individuals periodontally healthy and 20 periodontally diseased (10 females and 10 males in each group with mean age 48.6±11.1 and 49.6±10.2 respectively), sex and age- matched.	BOP, CAL and PPD. Diagnosis of CP: At least 50% of the teeth with PS ≥3mm, 10% bleeding on probing Control group: <10% of sites BOP and PPD <3 mm.	Values reported: Protein spots Outcomes: 344 proteins were identified in all samples, 20 proteins showed difference in abundance between groups, 19 of them in the periodontitis group. Main identified proteins: Protein S100-P, plastina-2, and neutrophil defensin.	Saliva collection: stimulated chewing a plain cotton role (for 1 minute, >0.7 mL). Quantitative and qualitative chemical analysis methods: Label-free quantitative LC-MS/MS.
Shin *et al*., 2018 [20] South Korea Deep sequencing salivary proteins for periodontitis using proteomics.	Cohort study 107 participants with periodontitis (36 females and 71 males, mean age 64.2± 9.0) and 100 healthy controls (35 females and 65 males, mean age 64.2± 9.3). 36 individual age, sex, and smoking matched.	ABL evaluated with panoramic radiographic. Diagnosis of periodontitis "incipient" in presence of proximal bone loss of ≥3 mm in ≥ 2non-adjacent teeth, "severe" in presence of proximal bone loss of ≥5 mm in ≥30% of teeth present.	Values reported: Protein spots Outcomes: 744 proteins were detected per sample. Main identified proteins: Proteins S100A8, S100A9 expressed highest value of total relative abundance, followed by zinc- alpha-2-glycoprotein, carbonic anhydrase 6, Ig mu chain C region.	Methods of saliva collection: Unstimulated (for 10 minutes). Quantitative and qualitative chemical analysis methods: LC-MS/MS, ELISA.
Bostanci *et al*., 2018 [21] Turkey Targeted proteomics guided by label-free quantitative proteome analysis in saliva reveals transition signatures from health to periodontal disease.	Case-control study. 17 individuals with chronic periodontitis (females and males, mean age ±) and 16 periodontally healthy (36 females and 71 males, mean age 64.2± 9.0).	BOP, CAL, PI, PPD and radiographic bone loss Diagnosis of CP: CAL ≥ 5mm in four non-adjacent teeth, PPD ≥ 6mm and 50% of alveolar bone loss. Periodontal health group: CAL ≤2mm and PPD ≤ 3mm and no detectable alveolar bone loss.	Values reported: μg/mL and fold change. Outcomes: Total proteins concentrations for periodontitis group were 764.3 μg/mL and 1140 μg/mL in healthy subjects, 67 proteins were different between groups. Main identified proteins: matrix metalloproteinase-9, ras-related protein-1, actin-related protein 2/3 complex subunit 5.	Methods of saliva collection: Unstimulated (for 5 minutes). Quantitative and qualitative chemical analysis methods: Label-free quantitative LC-MS/MS and LC-SRM.
Hartenbach *et al*., 2020 [22] Brazil Proteomic analysis of whole saliva in chronic periodontitis.	Case-control study. 10 subjects with periodontal health (7 females and 3 males, mean age 29.9±4.4) and 30 with chronic periodontitis (14 females and 16 males, mean age 42.0±2.6).	BOP, CAL, GI, PL, PPB and SC. Diagnosis of periodontitis: n/a Full-mouth clinical measurements, at 6 sites per tooth, except third molars.	Values reported: Fold change Outcomes: 223 proteins were analyzed in saliva samples. Main identified proteins: Periodontitis group had 9 exclusive protein spots (fatty acid-binding protein and phosphoprotein transfer protein) and 2 were identified in healthy periodontal subjects (serpin B4 and keratin type I).	Methods of saliva collection: Stimulated with parafilm (≥1mL). Quantitative and qualitative chemical analysis methods: LTQ orbitrap Velos.

ABL, alveolar bone loss; BOP, bleeding on probing; PD, periodontal diseases; CP, chronic periodontitis; CAL, clinical attachment level; GI, gingival index; PI, plaque index; PL, supragingival plaque; PPB, probing pocket depth; SC, supragingival calculus; N/A, not available; MSC, methods of saliva collection; STx, sample treatment; C, centrifuged; S, stored; S; I, immediately processed; NPI, no protease inhibitor.

**Table 3:** Characteristics and results of included studies before and after non-surgical periodontal therapy.

Author, year, country, title	Design and study groups	Definition and measures of periodontal status	Intervention	Study findings	
				PICO 2	PICO 3
Haigh *et al*., 2010 [23] New Zealand Alterations in the salivary proteome associated with periodontitis.	9 subjects (7 male and 2 female) with mean age 54.9 years).	Diagnosis of CP: At least 50% of the teeth with PS ≥ 3mm, 10% bleeding on probing.	Treatment: Routine root planning and cleaning. Interval time between two samples obtained: 69 to 512 days, with a median of 187 days.	Values reported: Protein spots and fold change. Outcomes: 126 proteins were identified in saliva samples, 15 proteins with altered abundance, 11 of them more abundant from individuals with active diseases. Main identified proteins: S100A6/A8/A9), prolactin-inducible protein, and parotid secretory protein.	Methods of saliva collection: Stimulated with parafilm bolus (5 mL). Quantitative and qualitative chemical analysis methods: 2D- gel electrophoresis, liquid chromatography, MALDI-TOF-TOF and LC-MS/MS.
Sharma *et al*., 2015 [24] India Alteration in salivary proteins following non-surgical periodontal therapy in generalized chronic periodontitis subjects.	15 subjects with chronic periodontitis (7 females and 8 males) and 15 periodontally healthy (4 females and 11 males) between 30 and 45 years).	BOP, CAL, GI, PI and PPD. Diagnosis of CP: ≥ 5 mm in at least 5 teeth and radiographic evidence of alveolar bone loss, measured at six sites per tooth.	Treatment: Single sitting of full mouth scaling and root planning. Interval time between two samples obtained: 4 elapsed weeks.	Values reported: mg/100 mL. Outcomes: The salivary proteins were significantly higher in periodontitis group (164 ± 31.13 mg/100 mL) compared with periodontally healthy individuals (107.07 ± 34.84 mg/100 mL), and decreased after 4 weeks received single setting of therapy (144.80 ± 36.14 mg/100). Main identified proteins: N/A.	Methods of saliva collection: Stimulated with paraffin wax (for 5 minutes, ~5–10 mL). Quantitative and qualitative chemical analysis methods: Biuret bicinchonic acid protein assay (BCA Kit).
Lira-Junior et al., 2021 [25] United Kingdom Levels of myeloid-related proteins in saliva for screening and monitoring of periodontal disease	72 subjects with chronic periodontitis and 60 periodontally healthy, between 18 and 65 years.	Diagnosis of CP: ≥8 teeth with ≥ 5 mm, and BOP ≥ 30%.	Treatment: Routine root planning and cleaning. Interval time between two samples obtained: 3 and 6 months.	Values reported: ng or pg/ mL. Outcomes: 7 myeloid-related markers proteins were identified in saliva samples of patients with periodontitis. Main identified proteins: CSF-1, S100A8/A9, S100A12, IL-1β, MMP-8 and HGF were significantly elevated in saliva.	Methods of saliva collection: Unstimulated Quantitative and qualitative chemical analysis methods: Immunoassays.

ABL, alveolar bone loss; BOP, bleeding on probing; PD, periodontal diseases; CP, chronic periodontitis; PTx, after periodontitis treatment; CAL, clinical attachment level; GI, gingival index; PI, plaque index; PL, supragingival plaque; PPB, probing pocket depth; SC, supragingival calculus; N/A, not available; CSF-1, colony-stimulating factor-1; IL-1β, Interleukin-1β, MMP-8, matrix metalloproteinase-8. MSC, methods of saliva collection; STx, sample treatment; C, centrifuged; S, stored; S; I, immediately processed; NPI, no protease inhibitor.

### Risk of bias

**Fig 2:**
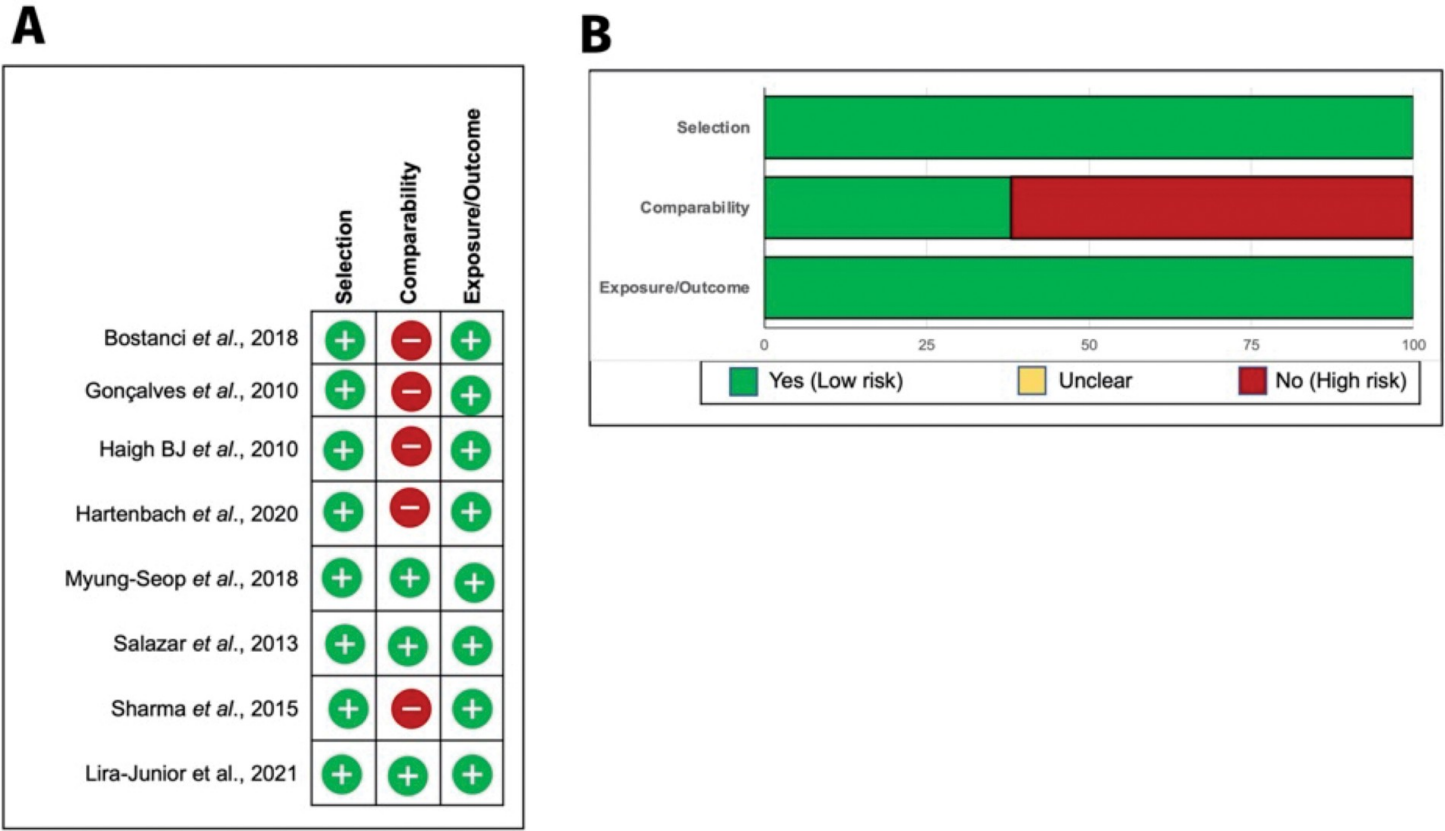
Assessment of risk of bias. Individual (A) and summarized (B) assessment of the risk of bias for included studies. +, low risk of bias; -, high risk of bias.

Risk of bias assessment was performed according to the Newcastle-Ottawa methodological quality scale using Review Manager version 5.4 (Fig 2). Among the eight included studies, three fulfilled all domains (37.5%) with a low risk of bias, whereas five studies (62.5%) presented high bias. In contrast, the quality was good for both assessment selection and exposure/outcomes. These biases were related to the representativeness of the cases/exposed cohort and comparability category. Based on the design or analysis, these domains evaluated the selection and comparability of cases and controls/cohorts.

### Reported proteins

The reported proteins were analyzed in the 8 eight selected studies. Seven studies (87.5%) reported the identification of proteins with relevance to the periodontal process. One of the studies described a high concentration of proteins in patients with chronic periodontitis (p<0.05) compared to healthy controls [24].

Since different protein access numbers were used to identify the proteins, we unified them with ExPASy (https://www.expasy.org/). In addition, the molecular and cellular functions of the major proteins in the saliva were categorized using the Ingenuity Pathway Analysis (IPA) and reviewed in UniProt (https://www.uniprot.org/) (Table 4).

**Table 4:** Main proteins in saliva with relationship to the inflammatory response.

Reference	[18]	[19]		[20]			[21]			[22]	[23]			[25]		
Protein access (Fold increase or p-value)	**P05062 (4.57)**	**P25815^ (2.4)**	**P13796 (2.2)**	**P59666 (2.1)**	**P05109^ (*p* = 0.043)**	**P06702^ (*p* = 0.014)**	**P25311 (*p* = 0.015)**	**P14780 (1.08)**	**P62834 (1.12)**	**Q01469 (*p<*0.05)**	**P01861 (*p<*0.05)**	**P05109^ (2.31)**	**P06702^ (1.99)**	**P05109^ P06702^ (*p<*0.001)**	**P01584 (*p<*0.001)**	**P09603 / Q6ZMJ4 (*p<*0.001)**
**Disease and disorders**																
Inflammatory response	*		*	*	*	*		*		*	*	*	*	*	*	*
Inflammatory disease			*	*	*	*		*			*			*		*
**Physiological system development and function**																
Hematological system development and function	*	*	*	*	*	*	*	*		*	*	*	*	*	*	*
Immune cell trafficking	*	*	*	*	*	*	*	*	*	*	*	*	*	*	*	*

The nomenclature used to name the proteins was unified with ExPASy (http://www.expasy.org). ^ S100 family members.

## Discussion

The present systematic review mined literature of the last twelve years to describe the main proteins in the saliva of subjects with chronic periodontitis. Some variations were detected in clinical parameters used in periodontal diagnosis. For example, only 25% of the studies reviewed, two articles, used the same criteria to identify the presence and absence of periodontal disease [19, 21].

### Altered proteins in chronic periodontitis

Of the eight studies analyzed in this study, four reported that members of the S100 proteins were the most representative groups of proteins, with an increase in patients with chronic periodontitis [19, 20, 23, 25]. However, Shin et al. reported a statistically significant difference in the quantified S100A8 in participants without periodontitis (430 pg/mL) compared to patients with periodontitis (11163 pg/mL) [20].

The S100 family members belong to a calcium-binding protein group with various intracellular and extracellular functions [8]. Most of the S100 proteins are related to biological processes such as leukocyte migration, inflammatory response, chemotaxis, and aggregation of neutrophils [26]. Additionally, the S1009 protein induces phagocytosis, increasing the bactericidal activity of human neutrophils, and is critical in controlling microbial infection [27]. On the other hand, the S1008 protein has also been reported to be a potential biomarker in saliva for oral squamous cell carcinoma diagnosis [28].

In the present study, two studies reported increased concentrations of S1008 and S1009 using the quantitative proteomics [23, 25]. In addition, another study showed that circulating monocytes from periodontitis patients had an altered expression of S100A12, suggesting its involvement in the pathogenesis of periodontitis [29].

The results in Table 2, which compared healthy periodontium with chronic periodontitis, showed differences attributable to study designs. For example, PICOS question 3 differed because of the saliva collection method and the use of protease inhibitors.

The non-surgical periodontal treatment requires a reduction of the subgingival biofilm since the procedure could be associated with tissue breakdown and the possible alteration of the composition and concentration of proteins during wound healing. A previous study reported that MMP-12 and S100/calgranulin concentration in saliva was stable and related to periodontal inflammation and adequate periodontal treatment [30]. Moreover, *Porphyromonas gingivalis*, an important pathogen bacterium that produces biofilm and is a keystone in periodontitis, enhanced expression of MMP 9 and interleukin-8 when grown in the culture of cell carcinoma. This event suggests the possibility of a direct relationship between orodigestive cancers and the *P*. *gingivalis* [31]. Therefore, we conclude that those biomarkers should measure the early detection and monitoring of an early stage of periodontitis.

### Altered proteins concentration before and after periodontal treatment

Three studies matched the eligibility criteria to compare the evolution of patients with periodontitis that received non-surgical treatment; however, only two studies showed the identification of the salivary proteins (Table 3) [23, 25]. These studies differ in the results because of the techniques used in quantifying proteins (mass spectrometry vs. immunoassays), the number of patients, and the method of collection (stimulated vs. unstimulated). Interestingly, results showed an up-regulation of colony-stimulating factor-1 (CSF-1), S100A8/A9, S100A12, interleukin (IL)-1β, matrix metalloproteinase (MMP)-8, and hepatocyte growth factor (HGF), in patients with periodontitis with a statistical significance of p<0.001 [25]. When these patients followed a non-surgical treatment, a reduction of IL-1β and MMP-8 was measured. Moreover, the ratio S100A8/S100A9 in saliva can differentiate distinct groups of periodontitis [25].

On the other hand, the second study used a quantitative biochemical parameter (bicinchoninic acid protein assay) to determine protein concentrations, but the identities of the proteins needed to be reported [24].

The differences in the methodology between the studies imply that the evidence needed to be sufficient to compare changes in protein profile before and after non-surgical periodontal therapy. Therefore, minimally invasive treatment is required to evaluate changes in the altered protein concentration before and after periodontal treatment. For example, a recent study with ultrasonic instrumentation combined with air polishing showed relevant results for the non-surgical treatment of periodontal pockets without complications [32]. Another example of successful and minimally invasive treatment is ozone water irrigation, which presents an adequate clinical evolution and a reduction of inflammatory mediators in the saliva [33]. In this context, evaluating these proteins in saliva could help assess oral hygiene status [34].

## Conclusions

Our systematic review results suggested that the salivary proteins profile is an early biomarker of periodontitis and early detection of other non-communicable diseases. Proteomics tools help elucidate and understand its changes under healthy conditions and the transition to periodontal disease. Furthermore, the family of S100 protein is a potential periodontitis biomarker and could help differentiate distinct subgroups of periodontitis. Thus, further high-quality studies are recommended; some of the study design’s main remarkable points should be considered, such as the saliva collection and use of protease inhibitors to stabilize the sample. Although the evidence between the studies implicated was insufficient to compare changes in salivary protein profile, well-designed studies showed statistical significance in protein concentration with potential like early biomarkers. Nevertheless, future research is needed to diagnose the disease’s early stages and monitor the effectiveness of treatment.

## Supporting information

S1 Checklist (DOCX)
